# Dynamic spatial patterns of leaf traits affect total respiration on the crown scale

**DOI:** 10.1038/srep26675

**Published:** 2016-05-26

**Authors:** Xiaolin Wang, Hongxuan Zhou, Fengsen Han, Yuanzheng Li, Dan Hu

**Affiliations:** 1 Research Center for Eco-Environmental Sciences, Chinese Academy of Sciences, State Key Laboratory of Urban and Regional Ecology (SKLURE), Beijing 100085, PR China

## Abstract

Temporal and spatial variations of leaf traits caused conflicting conclusions and great estimating errors of total carbon budget on crown scales. However, there is no effective method to quantitatively describe and study heterogeneous patterns of crowns yet. In this study, dynamic spatial patterns of typical ecological factors on crown scales were investigated during two sky conditions, and CEZs (crown ecological zones) method was developed for spatial crown zoning, within which leaf traits were statistically unchanged. The influencing factors on hourly and spatial variations of leaf dark respiration (*R*_*d*_) were analysed, and total crown respiration (*R*_*t*_) was estimated based on patterns of CEZs. The results showed that dynamic spatial patterns of air temperature and light intensity changed significantly by CEZs in special periods and positions, but not continuously. The contributions of influencing factors on variations of *R*_*d*_ changed with crown depth and sky conditions, and total contributions of leaf structural and chemical traits were higher during sunny days than ecological factors, but lower during cloudy days. The estimated errors of *R*_*t*_ may be obviously reduced with CEZs. These results provided some references for scaling from leaves to crown, and technical foundations for expanding lab-control experiments to open field ones.

The carbon budget of plants is crucial to the ecosystem carbon balance^1^,^2^,^3^, and crown accounts for most plant carbon budget^4^,^5^. Typically, the crown carbon budget was estimated by the summary of individual leaf gas-exchange with the assumption that the crown was uniform^6^,^7^,^8^,^9^. However, recent studies show that noteworthy crown heterogeneities appeared in several crowns resulted that leaf structural and functional traits greatly varies with crown positions^3^,^7^,^8^,^9^,^10^.

Heterogeneities of some ecological factors on a crown scale had been reported, especially in high trees. Light intensity (photosynthetic photon flux density, *I*) and air temperature (*T*) decline markedly with crown depth^3^,^11^, and *T* generally declines with crown depth due to within-crown shading^12^,^13^. However, there is currently little research on the spatial heterogeneity of relative air humidity (*RH*) and CO_2_ concentration (*C*_*air*_), which are also related to leaf traits. Leaf dry mass per area (*LMA*), leaf nitrogen and carbon content per area (*N*_*a*_ and *C*_*a*_) have been found to respond independently to crown position, which are essential for the analysis of heterogeneous crowns^6^,^14^,^15^,^16^,^17^. *LMA* and *N*_*a*_ decrease with crown depth mostly, which are considered to be related to the vertical light gradients in crowns, but the significant decline did not always occur^3^,^6^,^8^,^16^,^18^. *C*_*a*_ is due to the comprehensive effects of various crown factors, but very few studies have described its spatial heterogeneity^16^. Until now, the spatial heterogeneity of ecological factors, leaf structural and chemical traits still require further study.

Leaf dark respiration (*R*_*d*_) is a large fraction of the total carbon budget in plant from day to night, which is affected by environmental factors and leaf structural and chemical traits^14^,^15^,^16^,^19^,^20^,^21^. To our knowledge, the variation of *R*_*d*_ across the whole crown and contributions of influencing factors in diurnal course and crown positions still remain inconclusive largely due to a lack of quantitative and accurate research^5^,^16^,^21^. Total crown respiration (*R*_*t*_) account for up to two-thirds of total tree respiration^7^,^10^, the estimation of *R*_*t*_ on crown scale using gas-exchange technology fails to taking the heterogeneity of crown into consideration^22^,^23^. It is not convenient to estimate *R*_*t*_ and study the response of leaf traits to various environments on crown scales.

To solve the problems, it is crucial to describe and quantify heterogeneities of crown traits. Because of the various temporal and spatial patterns of crown traits, the spatial zoning of a crown is considered to be one better way to describe quantitatively spatial heterogeneities of crown traits. However, it is quite difficult to investigate lots of leaf gas-exchange traits automatically and continuously on crown scales in open field observations. Fortunately, many reports have suggested that leaf structural and functional traits were directly affected by ecological factors^3^,^5^,^24^,^25^,^26^. Temporal and spatial variations of ecological factors on crown scales can be obtained quantitatively. In view of this fact, variations of ecological factors were a solid foundation for spatial crown zoning. Through observations in open field, the diurnal dynamic patterns of typical ecological factors on a crown scale were surveyed. Reliable statistical techniques were used to quantitatively analyse the spatial significant differences of ecological factors. If there was no significant difference for any ecological factor between two adjacent crown areas, we considered the two areas as one “crown ecological zone (CEZ)”. Therefore, a CEZ was defined as a crown profile where ecological factors and leaf traits were unchanged statistically, and all CEZs were combined to be a complete coverage of a whole crown. Many researches indicated that ecological factors in different sky conditions (sunny or cloudy) were quite different^10^,^11^,^27^, thus, we assumed two different types of CEZs gradient patterns under two different sky conditions.

The heterogeneity of canopy traits does not always occur in all the crowns and all the time, in this study we took the crown of *Prunus lannesiana Wils*. as study target which is a common type of widely planted deciduous trees. Leaf structural and chemical traits and the diurnal dynamics of *R*_*d*_ across the crown during two sky conditions were analysed based on CEZs. We further explored the impact of a heterogeneous crown by calculating and comparing the *R*_*t*_ both in a heterogeneous crown and a “uniform crown”. We hypothesized that: (1) the crown could be zoned by CEZs, based on the dynamic patterns of *T* and *I* on crown scales, and the CEZs method is a sufficient and replicated method for spatial crown zoning to deal with heterogeneous crowns; (2) *R*_*t*_ is affected by dynamic patterns of leaf traits on crown scales, and the CEZs method of estimating *R*_*t*_ is reliable and practical compared to traditional ways; (3) the dominant influencing factors on variation of *R*_*d*_ are largely different between each pair of CEZs, during two sky conditions. In this paper, the traditional gas-exchange technology and spatial crown zoning method are combined to deal with the problem of scale transformation from leaves up to crown, and we emphasize the spatial variations of leaf traits on a crown scale. Additionally, we expect to achieve a better understanding of the impacts of typical ecological factors and leaf structural traits on *R*_*d*_ on a heterogeneous crown scale. This study will serve as a methodological reference for studying the response of the spatial heterogeneity of crown leaf respiration to local micro-climate changes.

## Results

### Temporal and spatial heterogeneity of ecological factors on a crown scale

**Figure 1:**
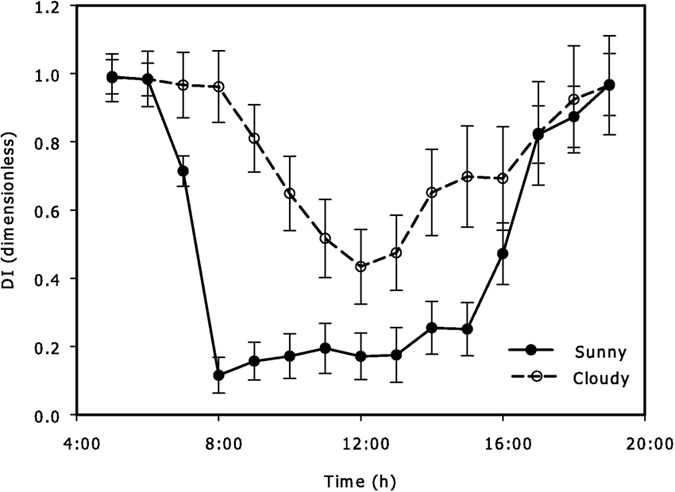
Diurnal changes in the incident diffuse index (*DI*) during sunny (●) and cloudy (○) days in summer. All values are means ± SE; *n* = 20.

**Figure 2:**
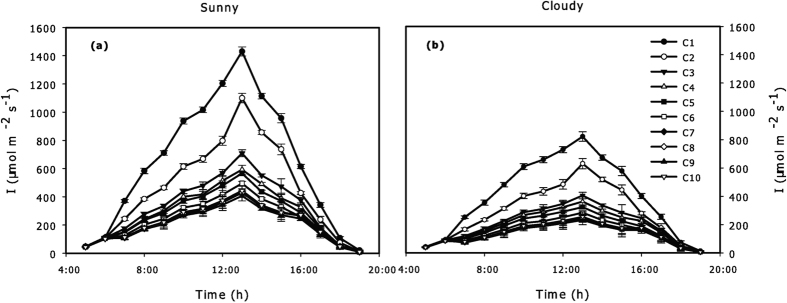
Diurnal changes in the incident photosynthetic photon flux density (*I*) in ten observed areas during sunny (**a**) and cloudy (**b**) conditions in summer. Different symbols are used to indicate different areas: ● = C1, ○ = C2, ▼ = C3, △ = C4, ■ = C5, □ = C6, ♦ = C7, ⋄ = C8, ▲ = C9, ▽ = C10. All values are means ± SE; *n* = 120.

The diffuse index (diffuse fraction of total radiation, *DI*) was higher at dawn (5:00–7:00) and dusk (17:00–19:00) than that during the rest of a day under two sky conditions (Fig. 1). *DI* during cloudy conditions was significantly higher than that during sunny conditions from 8:00 to 16:00 (Fig. 1) (p < 0.001). The vertical variation of *I* on a crown scale could be described with unimodal curves during two sky conditions, and the maximum *I* appeared at 13:00 (Fig. 2a,b). The fluctuating range of *I* during sunny days was 8.14–1430.40 μmol m^−2^·s^−1^, with an average of 301.39 μmol m^−2^·s^−1^, while during cloudy days, it was 5.48–821.98 μmol m^−2^·s^−1^, with an average of 192.69 μmol m^−2^·s^−1^ (Fig. 2a,b). The significance of *I* appeared between each pair of C1, C2, and C3 during sunny days (Fig. 2a), while it appeared between each pair of C1, C2, C3, and C4 during cloudy days (p = 0.005) (Fig. 2b). Hence, the spatial heterogeneity of the vertical variation *I* showed a 3-CEZs and 4-CEZs gradient pattern respectively under sunny and cloudy conditions (Supplementary Fig. S4).

**Figure 3:**
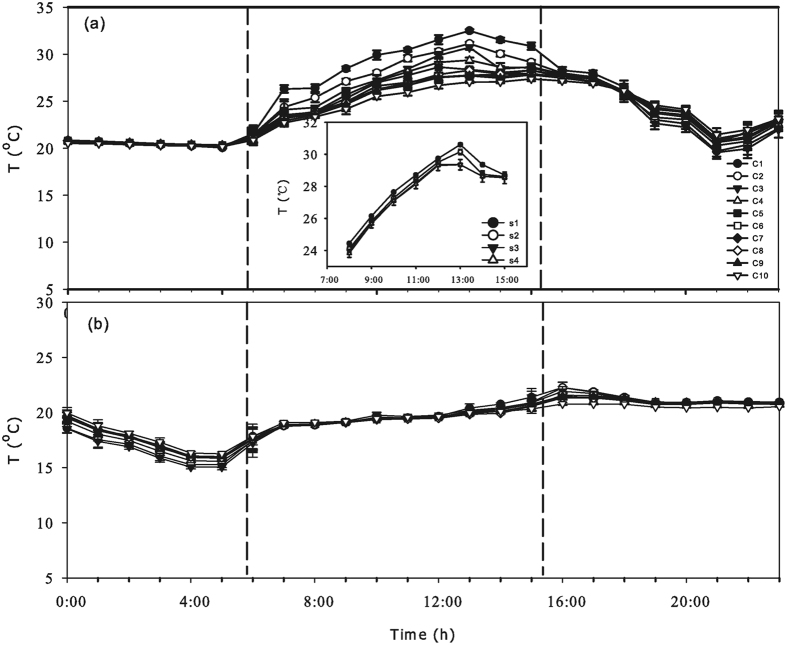
The hourly average air temperature (*T*) of ten observed areas (C1-C10) during sunny (**a**) and cloudy (**b**) conditions in summer. Different symbols are used to indicate different areas: ● = C1, ○ = C2, ▼ = C3, △ = C4, ■ = C5, □ = C6, ♦ = C7, ⋄ = C8, ▲ = C9, ▽ = C10. The inserted figure is hourly average T of sub-areas (s1, s2, s3, s4) at 8:00 to 16:00 during sunny condition. Different symbols are used in inserted figure to indicate different sub-areas: ● = s1, ○ = s2, ▼ = s3, △ = s4. All values are means ± SE; *n* = 36.

The hourly *T* showed a periodic change during sunny conditions, peaked at 31.4 °C in the meridian hour (13:00), and dropped down to 19.6 °C at 5:00 with an average of 24.1 °C (Fig. 3a). During cloudy conditions, a similar periodic change occurred in which the fluctuating range was 15.0–23.2 °C, peaked from 15:00 to 16:00 and dropped to the minimum at 5:00 (Fig. 3b). There were significant differences in *T* between each pair of the areas of C1, C2, and C3 from 8:00 to 15:00 (p = 0.004), while there was no significant difference between each pair of areas from C4 to C10 during sunny conditions. No significant difference was observed during the rest of a sunny day and all the cloudy day. From 12:00 to 15:00 during sunny conditions, there were significant differences in *T* between the sub-areas s1 and s2 (p = 0.005) but no significant difference between each pair of the sub-areas s2, s3, and s4 (Fig. 3a). Hence, the spatial heterogeneity of *T* showed a 4-CEZ gradient pattern during sunny conditions (Supplementary Fig. S5).

**Figure 4:**
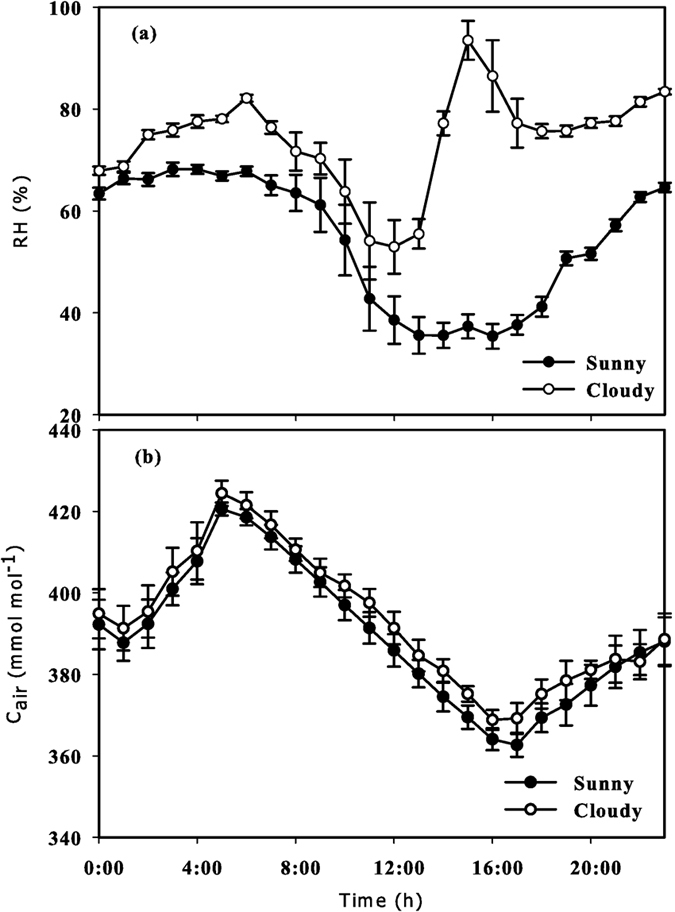
The hourly average air relative humidity (*RH*, **a**) and CO_2_ concentrations (*C*_*air*_, **b**) of the crowns under sunny (●) and cloudy (○) conditions in summer. All values are means ± SE; *n* = 36 (**a**) and 120 (**b**).

It can be seen that both the hourly *RH* and *C*_*air*_ showed periodic changes during the two sky conditions, but there were no significant differences in *RH* (Fig. 1a,b) and *C*_*air*_ (Fig. 1c,d) among the ten crown areas under the two sky conditions at our current temporal and spatial resolutions of observation. During sunny conditions, the fluctuating range of *RH* was 25.1–74.8% across the measurement period and peaked at 23:00, which lasted until 5:00 the next day; the minimum of *RH* appeared at 14:00 (Fig. 4a). During cloudy conditions, the fluctuating range of *RH* was 35.8–99.5%, the maximum *RH* appeared at 16:00 and 23:00, and the minimum *RH* also appeared at 14:00 (Fig. 4a). The *C*_*air*_ peaked at 5:00 and then dropped to the minimum at 17:00 under the two sky conditions. The *C*_*air*_ of the crown areas fluctuated between 358.2 and 423.0 μmol·mol^−1^ during sunny conditions and fluctuated between 363.5 and 428 μmol·mol^−1^ during cloudy conditions (Fig. 4b).

Therefore, it came up with a spatial crown pattern of ecological factors in a vertical direction based on the spatial heterogeneity of *T* and *I* under sunny and cloudy conditions. The spatially heterogeneous patterns of ecological factors divided the whole space of the crown into four different CEZs (D1, D2, D3, D4) during sunny conditions and into another four CEZs (E1, E2, E3, E4) during cloudy conditions (Supplementary Fig. S7).

### Variation of *LMA, C*_*a*_, *N*_*a*_, and *R*_*d*_between CEZs

**Figure 5:**
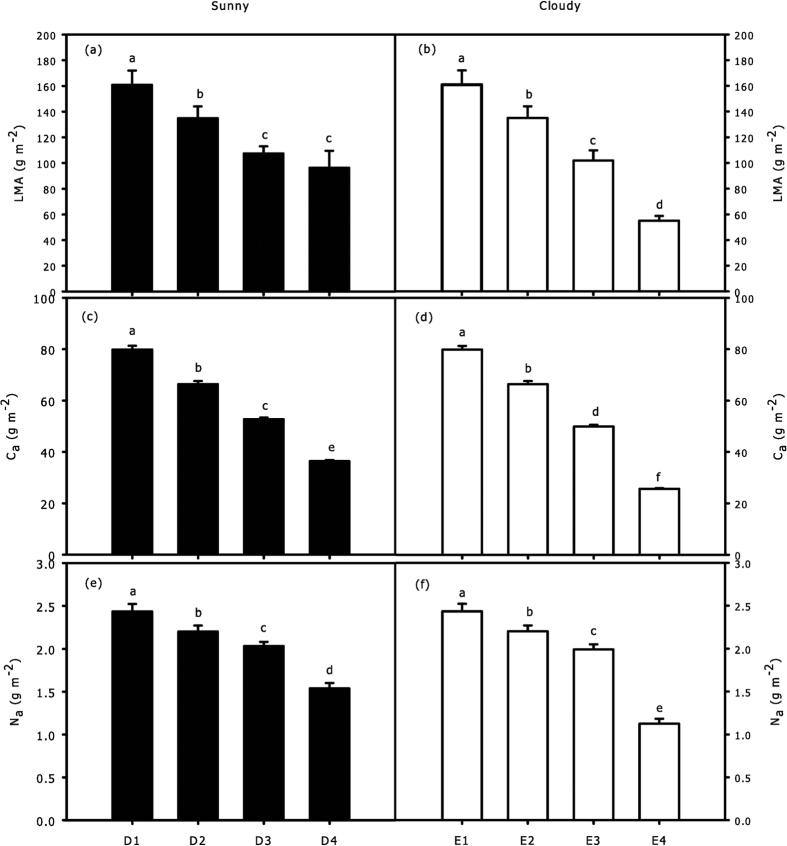
Variation of *LMA* (**a,b**), *C*_*a*_(**c,d**) and *N*_*a*_(**e,f**) of leaves between each pair of CEZs (D1, D2, D3, D4 under sunny condition and E1, E2, E3, E4 under cloudy condition) on a crown scale. Identical letters indicate homogeneous groups with statistically insignificant differences (p > 0.05). All values are means ± SE; *n* = 30.

*LMA* on a crown scale decreased with crown depth, and there were significant differences between each pair of the four CEZs (p = 0.005) in the sunny pattern except for D3 and D4 (Fig. 5a); there were significant differences between each pair of the four CEZs in the cloudy pattern (p = 0.006) (Fig. 5b). There were significant differences in *C*_*a*_ and *N*_*a*_ between each pair of the CEZs during two sky conditions (p = 0.004) (Fig. 5c,d,e,f).

**Figure 6:**
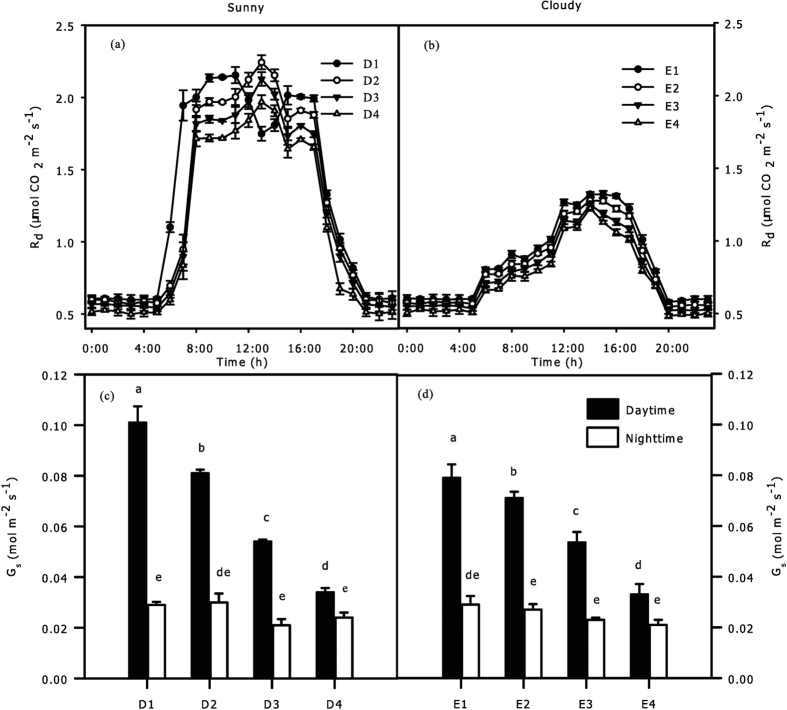
Spatial variations of leaf respiration rate (*R*_*d*_, **a,b**) stomatal conductance (*G*_*s*_, **c,d**) on a crown scale during sunny (**a,c**) and cloudy (**b,d**) conditions in summer. The hourly *R*_*d*_ was shown at every hour (**a,b**), ● = D1 and E1, ○ = D2 and E2, ▼ = D3 and E3, △ = D4 and E4. *G*_*s*_ of daytime (closed bar) was the average stomatal conductance in days, and *G*_*s*_ of night-time (open bar) was the average in nights. All values are means ± SE; *n* = 18.

*R*_*d*_ changed rapidly in the sunny days with a fluctuating range of 0.499–2.243 μmol CO_2_ m^−2^·s^−1^ and became gentle in the cloudy days with a fluctuating range of 0.483–1.321 μmol CO_2_ m^−2^·s^−1^ (Fig. 6a,b). In sunny days, *R*_*d*_ peaked at 12:00 to 13:00 in the CEZs of D2, D3, and D4, while it peaked at 11:00 in the CEZs of D1 and then dropped to a minimum at 3:00 to 5:00 (Fig. 6a); there were significant differences between each pair of the four CEZs (D1, D2, D3, D4) in every hour from 8:00 to 19:00 (p = 0.004), except the CEZ of D1 from 12:00 to 14:00, but there was no significant difference during the rest of the day. In cloudy days, the *R*_*d*_ peaked at 14:00 to 15:00 and then dropped to a minimum at 3:00 to 5:00; the significant differences between each pair of four CEZs (E1, E2, E3, E4) only occurred from 10:00 to 18:00 (Fig. 6b) (p = 0.01). The stomatal conductance (*G*_*s*_) appeared similar changes between CEZs under two sky conditions (Fig. 6c,d). *G*_*s*_ in the daytime considerably decreased with the depth of CEZs, while there was no obvious difference between each pair of CEZs in the nighttime.

### Contributions of ecological factors and leaf traits to the hourly and spatial variations of *R*_*d*_

**Figure 7:**
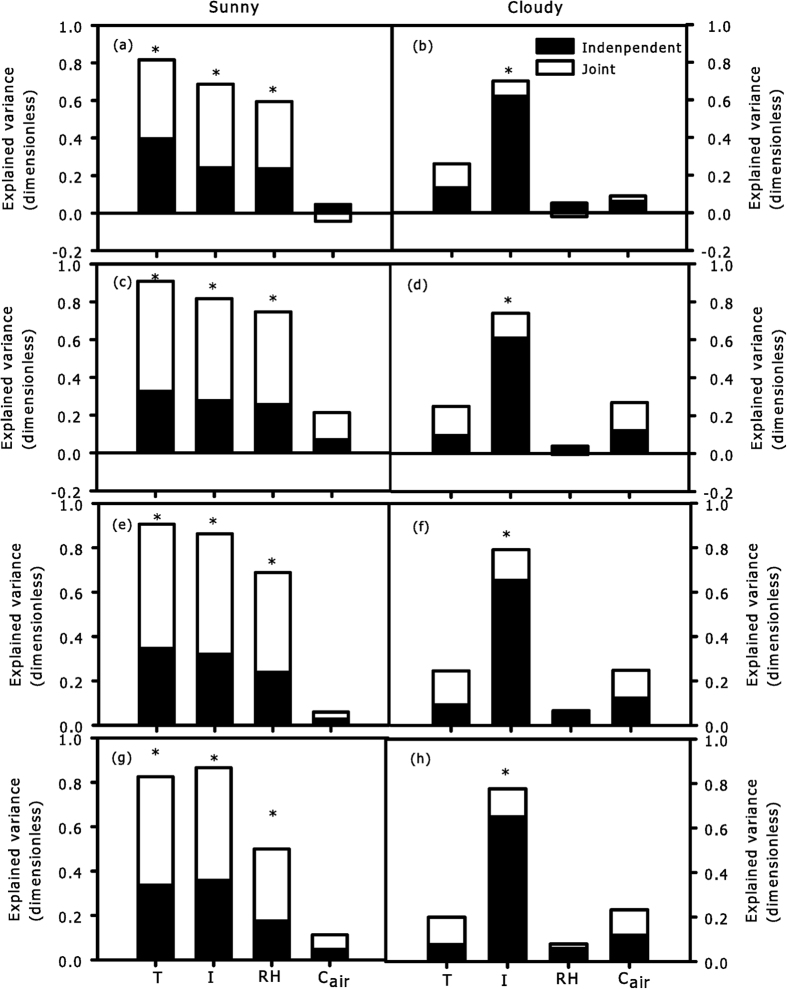
The independent (closed bar) and joint (open bar) explanatory variance of ecological factors for hourly *R*_*d*_in each CEZ during sunny (**a,c,e,g**) and cloudy (**b,d,f,h**) conditions as estimated by hierarchical partitioning, in which ‘*’ denotes that the independent effect due to this variable was significant at p < 0.05.

**Figure 8:**
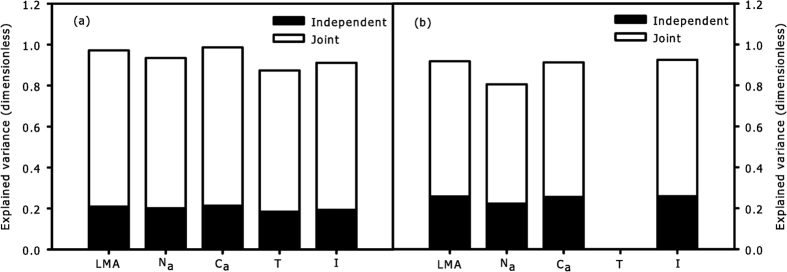
The independent (closed bar) and joint (open bar) explanatory variance of the factors for spatial *R*_*d*_ during sunny (**a**) and cloudy (**b**) conditions, as estimated by hierarchical partitioning, in which ‘*’ denotes that the independent effect due to this variable was significant at p < 0.05.

In sunny days, the independent effects of the explanatory variables *T*, *I* and *RH* were significant for the change of hourly *R*_*d*_ in each CEZ (Fig. 7a,c,e,g), while *T* had the highest independent and total (independent + joint) contributions among ecological factors (Fig. 7a,c,e,g); all the explanatory variables had higher joint contributions than independent contributions during sunny conditions (Fig. 7a,c,e,g). In cloudy days, the independent effect of *I* was significant for hourly variation of *R*_*d*_ in each CEZ, while other variables were not significant (Fig. 7b,d,f,h); *I* had a higher independent contribution than the joint contribution in each CEZ (Fig. 7b,d,f,h). The independent contributions of *T* decreased with the depth of CEZs, while the independent contributions of *I* increased with the depth of CEZs under both sky conditions (Fig. 7). *LMA*, *N*_*a*_, and *C*_*a*_ had higher contributions on spatial variations of *R*_*d*_ than ecological factors (*T* and *I*) under sunny conditions, and *C*_*a*_ had the highest total contribution on spatial variations of *R*_*d*_ among all variables (Fig. 8a). In contrast, *I* had higher contributions than leaf structural and chemical traits (*LMA*, *N*_*a*_, and *C*_*a*_) under cloudy conditions (Fig. 8b). Thus, the relative importance of variables for the spatial variation of *R*_*d*_ was different between two sky conditions.

### Estimation of crown respiration in CEZ patterns and a uniform crown

**Figure 9:**
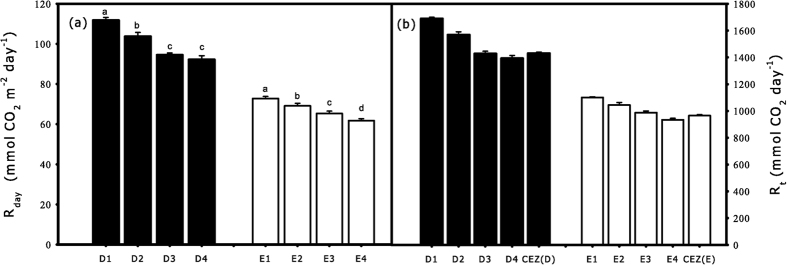
The cumulative respiration rates (*R*_*day*_) of CEZs (D1, D2, D3, D4, and E1, E2, E3, E4) and total crown respiration (*R*_*t*_) estimated by a uniform *R*_*d*_and CEZs method on crown scales. *R*_*day*_ and *R*_*t*_ were shown under sunny (closed bar) and cloudy (open bar) conditions, CEZ(D) and CEZ(E) were *R*_*t*_ estimated by using CEZs method respectively under sunny and cloudy conditions. Identical letters indicate homogeneous groups with statistically insignificant differences (p > 0.05). *n* = 6.

The cumulative respiration rates (*R*_*day*_) of CEZs were higher in sunny conditions than cloudy conditions (Fig. 9a), and *R*_*day*_ were significantly different between each pair of CEZs under both sky conditions (p < 0.001), except between D3 and D4. The CEZ-based *R*_*t*_ was lower than D1-based (when considering the crown as uniform for D1) and D2-based *R*_*t*_, while it was higher than D3-based and D4-based *R*_*t*_ during sunny conditions (Fig. 9b). The CEZ-based *R*_*t*_ was lower than E1-based, E2-based, and E3-based *R*_*t*_, while it was higher than E4-based *R*_*t*_ during cloudy conditions (Fig. 9b).

## Discussion

The aim of the study presented here was to explore a technological method to quantitatively describe the heterogeneity of a crown, and apply the method to study crown-dependent variation of leaf traits and estimate *R*_*t*_ more accurately. The aim was reached in defining CEZs across the crown based on the dynamic diurnal variations of ecological factors. All the measured ecological factors in a CEZs were statistically unchanged in any time of the day, and leaf morphologies (Supplementary Fig. S11) and functional traits were considered to be stable in the same CEZ, assuming that they were hardly affected by growth-regulating substances.

### The CEZs based on temporal and spatial heterogeneity of *T* and *I*

According to our results, there were no significant differences in the hourly *RH* and *C*_*air*_ during the two sky conditions (Fig. 4). The spatial variations of *T* and *I* appeared in special periods of a day and between some special areas (Figs 2 and 3). The *T, RH, C*_*air*_ and *I* in a horizontal orientation measured in each area had no significant difference regardless of areas and sky conditions (Supplementary Fig. S7). Therefore, *T* and *I* were the main reference indicators for spatial crown ecological zoning.

The spatial patterns of *T* and *I* was different under the two sky conditions (Figs S5 and S6) based on the statistical significances among areas and sub-areas; the property of incident light was a major factor underlying this phenomenon. During sunny conditions, leaves were illuminated from a single direction, which tended to be divided into brightly illuminated and heavily shaded areas^10^,^11^,^28^. Under diffuse conditions, irradiance illuminating into a crown comes from a larger fraction of the sky hemisphere, so shading is minimized. Thus, crowns are more evenly illuminated under diffuse irradiance than direct irradiance^10^,^11^. In our results, the *DI* during cloudy conditions was significantly higher than that during sunny conditions for most of the day (Fig. 1).

Leaf temperature changes with air temperature simultaneously, and the correlation between them is extremely notable; thus, *T* is a good indicator of leaf temperature^29^. The diurnal period with significant differences for *T* (9:00–15:00) was consistent with the period with a higher value of *I* in sunny days. Consequently, the hourly *T* changed almost simultaneously with *I*. However, *T* was also affected by physiological processes and airflow within a crown^30^,^31^,^32^; thus, the vertical variations of *T* might be more sensitive. As such, it appeared two significant parts in sub-areas due to the statistical significances of *T* (Fig. 3a). During cloudy conditions, weak heating rate of low irradiance and heat loss caused by airflow within a crown might result in non-significant variation of *T*, the spatial difference in *T* on a crown scale was not obvious (Fig. 3b). Thus, the spatial pattern of *T* was similar to that of *I*, and both were not changed continuously, but discretely by CEZs in term of statistics.

### Variations of *R*_*d*_ between CEZs on a crown scale

There was no actual consensus about how *R*_*d*_ varies with crown depth in spite of many reports on heterogeneous crowns^6^,^20^. Our results showed that the significant differences of *R*_*d*_ in different CEZs only appeared in special periods of the day, which were also different between the two sky conditions (Fig. 6a,b). Hence, we considered that time and position of sampling caused the inconsistent conclusions of current studies, excluding variations in species. Despite the fact that *T* directly affects *R*_*d*_, our investigation showed that *R*_*d*_ at predawn was very low but sharply rose after day break (Fig. 6a,b), while *T* did not dramatically change in this period (Fig. 3). Leaf stomata are the channels for CO_2_ and O_2_, and their behaviours are greatly affected by light^33^; thus, the spatial variation of leaf stomatal conductance (*G*_*s*_) might be the reason for the conflict. Figure 6b showed that *G*_*s*_ was much lower in the night-time than daytime (p < 0.01), and there were nearly no significant differences between each pair of CEZs at all night-time periods in this crown. Tarvainen *et al*. showed that *G*_*s*_ was the main reason for differences in gas exchange between daytime and night-time, and the low value of *G*_*s*_ resulted in no difference in gas exchange in crown positions^34^. Hence, the limited *G*_*s*_ was the reasons for the lower *R*_*d*_ and the non-significant differences among CEZs in night-time. Furthermore, the temporary decline of *R*_*d*_ from 11:00 to 13:00 in sunny days might be due to stomata closure at noon.

### The study of influencing factors of hourly and spatial variations of *R*_*d*_ using CEZs

To clarify the influencing factors on spatial variation of *R*_*d*_ on the crown scale, the vertical variation of *R*_*d*_ and *R*_*d*_-related factors should be described firstly. Leaves from one CEZ were substantially different from other CEZs in *R*_*d*_ and some related factors. Although leaves from plenty of sampling positions might greatly covered difference, lots of repetitions are needed to obtain obvious gradients of leaf traits across the crown. Moreover, the investigations of hourly variation of *R*_*d*_ need to be carried out in a constant *R*_*d*_. The hourly variation of *R*_*d*_ can be investigated in one CEZ, and the influencing factors of that can be compared between the CEZs. In this study, the CEZs method took full use of the advantage of dynamic pattern of canopy traits to study the contributions of influencing factors on temporal and spatial variation of *R*_*d*_ on the crown scale.

Tissue constructions need a period for accumulation, so we assumed there were no significant differences in *LMA*, *N*_*a*_ and *C*_*a*_ between every hour, the ecological factors were the only direct factors influencing the change of hourly *R*_*d*_. However, a long period of variations in *T* and *I* could cause differences in leaf structural traits of different CEZs (Fig. 5); thus, the spatial heterogeneity of *R*_*d*_ between CEZs was affected by ecological factors and leaf structural traits. In this case, the results of hierarchical partitioning showed that *T, I* and *RH* had significant independent contributions on the hourly variations of *R*_*d*_during sunny conditions (Fig. 7a,c,e,g), but only *I* during cloudy conditions (Fig. 7b,d,f,h). Light intensity decreases severely with depth of crown, and light is considered to be the key factor of leaf traits in lower crowns^3^,^35^. Due to thermo-regulation of leaves, lower crowns with higher leaf density could be less sensitive than upper crowns. As a consequence, the independent contributions of *T* tended to be higher in upper crown than that in lower crown, while the independent contributions of *I* tended to be higher in lower crown during two sky conditions (Fig. 7). None of explained variance had significant independent contribution to spatial variation of *R*_*d*_, and a far greater portion of the explained variance was related to the joint contributions (Fig. 8). As such, the interaction of multiple factors played a more important role in influencing the spatial variation of *R*_*d*_. In addition, *C*_*a*_ more comprehensively reflected the response of leaves to ecological factors^16^, *C*_*a*_ had the highest total contribution to spatial variations of *R*_*d*_ of all variables (Fig. 8), which further confirms that variation of *R*_*d*_ was affected more by the joint rather than the independent effects of the factors.

### Variation of *R*_*t*_ on the crown scale with the help of CEZs

Many studies attempted to extrapolate the gas-exchange parameters to crown level, and the accuracy of a method depended on the sampling positions of the crown^36^,^37^. In the previous reports, sampling positions were selected by the uniform method, such as the “big-leaf model”^38^. The positions of the crown affect *R*_*d*_ and its response to ecological factors on the crown scale^3^,^7^. Our results demonstrated that crown position, sampling time and sky conditions could have significant effects on *R*_*d*_ (Fig. 6a,b). The multilayer model has taken leaf variations into account, it fails to utilize these variations. Furthermore, the sampling positions are selected by using the normalized Gaussian distances, although the mathematical accuracy could be admitted, it fails to select the representative positions^39^. The CEZs in this study provided better reference systems of notable significance in leaf traits, and the positions from all the CEZs were considered good representations of the crown. Compared to the CEZs method, the *R*_*t*_ were overestimated when selecting positions in upper and middle crown (CEZs of D1, D2, E1, and E2), and underestimated when selecting positions in lower crown (CEZs of D3, D4, E3, and E4) (Fig. 9b). Furthermore, simplifying assumptions regardless of sky conditions tended to result in an overestimation or underestimation of the actual *R*_*t*_ (Fig. 9b). Thus, the CEZs method which had taken sky conditions and the spatial patterns of the crown into consideration can reduce the estimation errors of *R*_*t*_.

### Feasibility and simplicity of the CEZs method

Two 4-CEZ gradient patterns reasonably reflected the spatial patterns of ecological factors and leaf traits and reduced the errors in estimating *R*_*t*_ to the greatest possible extent. However, ecological factors and leaf traits on a crown scale are affected by climate, seasons, species, and crown types. By the same technology, we investigated the CEZs gradient patterns of the selected trees in spring and autumn (Supplementary Fig. S9), and other trees of different species (Supplementary Fig. S10) to indicate the applicability of the method. The CEZs were similar in all the investigated trees, although the patterns of CEZs were not always the same. Due to the same CEZ gradient patterns for a crown of *Prunus lannesiana Wils*., the estimation on *R*_*t*_ in autumn was consistent with that in summer (Supplementary Fig. S12). And the contributions of ecological factors and leaf traits on the hourly and spatial variation of *R*_*d*_ in autumn were nearly the same with that in summer because of the constant leaf structural and chemical traits in full-developed crowns (Supplementary Figs S13 and S14).

It was worth noting that the height of D3 was only 2.5% of the whole crown, and the *R*_*day*_ of D3 was non-significant with D4. In addition, D3 occurred from 12:00 to 15:00 in a day, while other CEZs occurred from 8:00 to 15:00. For most of the day, ecological factors of D3 showed the same variation with D4 except for 12:00–15:00. Furthermore, the sub-dividing areas by 0.1m is beyond the level that most models would require, and undetectable in field collections. Thus , in field applications, we suggest combining D3 and D4 under sunny conditions. Leaf traits of the lower percentage of 80% (sunny days) and 70% (cloudy days) were statistically unchanged in our studies, which was consistent with non-significance of *T*, *I*, *LMA*, and *N*_*a*_ between lower crown areas reported in numerous studies^10^,^21^,^40^. Meanwhile, more accurate scales of observation might generate more convincing results and help to identify much more defined boundaries of CEZs.

## Conclusions

With this study, the temporal and spatial heterogeneities of ecological factors (*T*, *I*, *RH*, *C*_*air*_) in *Prunus lannesiana Wils*. were clarified on the fully-developed crown scale in north temperate regions (Beijing): the vertical variations of *T* and *I* occurred in special periods of a day, and were different between two sky conditions. *T* and *I* were not continuously decreased with depth of crown, but decreased discretely by CEZs, even if the observational accuracy of the crown decreased to 0.1m. With the fact of temporal-spatial features of *T* and *I* in the crown scale, it came up with CEZs gradient patterns which were used to indicate the spatial patterns of a crown. The CEZs method provides a system of crown with obvious inter-zones differences and intra-zone homogeneity. With the help of CEZs, the contributions of influencing factors on hourly variations of *R*_*d*_ of *Prunus lannesiana Wils*. and their relationship with depth of crown were analysed for the first time, and the contributions of fully-developed leaf morphologies on spatial variations of *R*_*d*_ were compared with ecological factors. The estimated values of *R*_*t*_ in north temperate regions (Beijing) were affected by dynamic spatial patterns of leaf traits (*LMA*, *N*_*a*_, *C*_*a*_, *R*_*d*_) on crown scales, which could be displayed clearly using CEZs method, thus, estimation of *R*_*t*_ with CEZs method was proved to be less errors than previous methods.

Our results may help to establish effective technical methods to estimate total crown respiration and better understand spatial response of ecological factors and leaf traits of a crown to environmental conditions in north temperate regions (Beijing).

## Materials and Methods

### Field sites and plant materials

This experiment was conducted in an urban green space in Haidian District, Beijing City of China during July-September 2014. Beijing is located in a northern temperate zone from115.7°E-117.4°E, 39.4°N-41.6°N. The experimental site was a monsoon-influenced humid continental climate with a hot and humid summer with the mean air temperature of 24.4 °C and precipitation of 503.1 mm. The sunshine duration during July-September was 230–245 hours per month. The site was a typical plant community primarily composed of trees, shrubs and herbaceous plants. The study site was not blocked by high buildings or large trees. The selected trees of *Prunus lannesiana Wils*. were described in Supplementary Table S1. The sky conditions were determined with the following method: 1) the sunny and cloudy days were selected according to weather reports by China Meteorological Administration. 2) visual observation during experimental period was used to exclude the diurnal temporary changes of sky condition. The cloudy sky condition was defined as the solar disc is invisible^41^, and there is no clear bonder of shade in the crown. 3) besides, in many ecological studies, the diffuse index (diffuse fraction of total radiation, *DI*) was used to separate days into sunny and cloudy, *DI* mostly remained less than 0.27 during sunny days while more than 0.65 during the sunny and cloudy days, respectively^10^,^41^,^42^,^43^. Microclimatic characteristics during sunny and cloudy conditions were measured and are shown in Supplementary Table S2.

### Ecological factors on a crown scale

We took a range of 4.0 m heights of the crown as survey areas, covering almost all the areas of a crown based on the tree’s structural parameters while taking into account the precision and resolution of the detectors. The survey areas were artificially divided into ten sequential areas (labelled as C1, C2, C3, C4, C5, C6, C7, C8, C9, C10) in a vertical direction where C1 was the 90–100% relative height of the whole crown, and each area contained 10% range of crown and sufficient leaves to measure leaf traits. The details of this technique are described in Supplementary Information (Supplementary Fig. S1).

The spectral composition of light transmitted into the crown and the hourly *I* (over the wavelength of 400–800 nm) in the crown areas was observed by using a fibre optic spectrometer equipped with a cosine corrector (AvaSpec-ULS 2048XL, Avantes, Netherland) in each area (C1-C10), from 5:00 to 19:00. In each area, we randomly performed sampling of 20 crown points and calculated the average of the observational data of six days during the two sky conditions.

The *T* and *RH* of each area were observed with sensors of air temperature and relative humidity, and were recorded with data loggers (Onset HOBO Data Loggers, Pocasset, Massachusetts, USA). The logging interval was 1 min, and the obtained data were averaged every 10 min. Thus, 6 numerical values for each sensor were acquired in one hour. According to the statistical results, much smaller intervals of sampling needed to be performed to distinguish the variation of *T* in the C3 area. Thus, we divided the area of C3 equally into four sub-areas (s1, s2, s3, s4); each sub-area was 0.1 m height (Supplementary Fig. S2). We also took an average of the six-day observational data of the variables *T* and *RH* during the two sky conditions.

The *C*_*air*_ was measured by a portable open-flow gas-exchange system (Li-6400, Li-Cor Inc., Lincoln NE, USA) that logged every hour in each area. We randomly performed sampling of 20 crown points and took an average of the observational data of six days during the two sky conditions.

The values of the variables *I*, *T*, *RH* and *C*_*air*_ in a crown horizontal orientation were also measured in each vertical area and sub-area, and the statistical significance was analysed using the same method as for the vertical orientation (Supplementary Fig. S4).

### Leaf traits on a crown scale

*LMA* was measured on leaves randomly sampled in each CEZ before dawn (4:00). Leaves were scanned using a leaf area meter (Li-3100, LI-COR, Inc.) to measure surface areas, then placed into a drying oven. After 15 min at 105 °C, leaves were dried at 85 °C until the weight no longer changed. LMA was calculated as a unit area of dry weight. We collected 50 samples in each CEZ. *C*_*a*_ and *N*_*a*_ were acquired with an elemental analyser (Vario EL III, Elementar, Germany) using powdery leaves; nine samples were collected in each CEZ.

Measurements of *R*_*d*_ were made with a portable open-flow gas-exchange system (Li-6400, Li-Cor Inc), and a workbench was used in high positions. Environmental conditions within the cuvette were controlled to match the measured air conditions. Leave of each CEZs were dark acclimated for a minimum of 20 min before measurements began, and the measurement of respiration was recorded only when gas exchange had equilibrated (taken to be when the rate of CO_2_ efflux was stable and the coefficient of variation for CO_2_ partial pressure differential between the sample and reference was <1%). The *R*_*d*_ of each CEZ was measured at 1-hour interval, and all the collected leaves were fully self-expanded and of a similar age.

### Estimation of crown respiration

The *R*_*t*_ of each zone was calculated from equation (1), where *R*_*day*_ was the cumulative respiration rates of each CEZ throughout the day, and calculated by trapezoidal integration over *R*_*d*_ in 24 hours by equation (2); *S*_*n*_ was the estimated area of a single leaf, which is the average of leaf areas in a CEZ. Number of leaves in unit volume was calculated, *N* was the number of all leaves in a CEZ, and was estimated with the total volume of a CEZ, assuming that the CEZ was a round estrade.


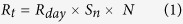



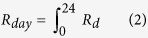


Therefore, the *R*_*t*_ based on CEZs was the summary of the *R*_*t*_ of CEZs, and the *R*_*t*_ based on a ‘uniform crown’ was calculated by using the constant *R*_*d*_ of a certain CEZ.

### Statistical analysis

The observed values of ecological factors at every hour were analysed statistically with one-way ANOVA in R-software (R 3.0.3: A programming environment for data analysis and graphics ^©^ 2014) to calculate the statistical significance of the difference among areas (confidence band of 95%). The significance of the differences in leaf traits between CEZs was analysed statistically via one-way ANOVA in R-software. Correlation analysis were made with the related modules in R-software.

The hierarchical partitioning method in R-software was used to identify the individual variables that significantly affected *R*_*d*_ in a crown. This method could provide a calculation of the independent and joint contribution with all other variables. This analysis was completed using the ‘hier.part package’ version 5.1^44^ that was a part of the R statistical package. Hierarchical partitioning depends on monotonic relationships between the response and predictor variables^45^,^46^. Hence, the data were log transformed to improve the linearity of relationships between *R*_*d*_ and other variables. Statistical significance was accepted at the upper 95% of confidence limit^44^. The graphics software SigmaPlot (Version 10.0) was used to create artwork.

## Additional Information

**How to cite this article**: Wang, X. *et al*. Dynamic spatial patterns of leaf traits affect total respiration on the crown scale. *Sci. Rep*. **6**, 26675; doi: 10.1038/srep26675 (2016).

## Supplementary Material

Supplementary Information

Supplementary Table S3

Supplementary Table S4

Supplementary Table S5

Supplementary Table S6

Supplementary Table S7

Supplementary Table S8
