# Supplementary material for: Dynamic spatial patterns of leaf traits affect total respiration on the crown scale
Source: Sci Rep. 2016 May 26;6:26675. doi: 10.1038/srep26675 (PMC4881035; doi:10.1038/srep26675)
Supplement: Supplementary Information [file srep26675-s1.pdf]

Supplementary Information

Dynamic spatial patterns of leaf traits affect total respiration on the crown scale

Xiaolin Wang[1], Hongxuan Zhou[1], Fengsen Han[1],Yuanzheng Li[1], Dan Hu[*1]

[1]Research Center for Eco-Environmental Sciences, Chinese Academy of Sciences, State Key Laboratory of Urban and Regional Ecology (SKLURE), Beijing 100085, PR China

Corresponding author:

Dan Hu

Tel: +86 10 62849199

Email: hudan@rcees.ac.cn

Address: 18 Shuangqing Road, Haidian District, Beijing, 100085

People's Republic of China

Supplementary Index

Fig. S1 One of the tree profiles for vertical observation of ecological factors and leaf structural traits on a crown scale.

Fig. S2 One of the tree profiles for vertical observation of air temperature (*T*) in the area of C3 on a crown scale.

Fig. S3 Diameter of temperature sensors.

Fig. S4 One of the top views of horizontal observation of ecological factors in each vertical area on a crown scale.

Fig. S5 An ecological zoning for a spatial gradient pattern in a vertical direction for variations of the photosynthetic photon flux density (*I*) on a crown scale under sunny (a) and cloudy (b) conditions.

Fig. S6 An ecological zoning for a spatial gradient pattern in a vertical direction for variations of temperature (*T*) on a crown scale.

Fig. S7 One of the hourly average values of *RH* (a,b), C_{air} (c,d), *T* (e,f) and *I* (g,h) for the four crown horizontal quadrants (H1, H2, H3, H4) in each vertical area (C1-C10) during sunny (a,c,e,g) and cloudy (b,d,f,h) conditions in summer. Each value was the average of five crown samplings from the same quadrant. All values were means ± SE. *n*=5.

Fig. S8 Ecological zoning integration of ecological factors for spatial gradient patterns on a crown scale under sunny (a) and cloudy (b) conditions.

Fig. S9 The CEZs gradient patterns of *Prunus lannesiana Wils* in spring (a,b) and autumn (c,d). The CEZs of the tree were shown under sunny (a,c) and cloudy (b,d) conditions separately.

Fig. S10 The CEZs gradient patterns of *Prunus cerasifera* (a,b) and *Koelreuteria paniculata* (c,d) in summer. The CEZs of the tree were shown under sunny (a,c) and

cloudy (b,d) conditions separately.

Fig. S11 Vertical variations in among different crown areas (closed bar) and sub-areas (open bar). Identical letters indicate homogeneous groups with statistically insignificant differences (p>0.05). All values are means ± SE; n=30.

Fig. S12 The cumulative respiration rates (R_{day}) of CEZs (D1, D2, D3, D4, and E1, E2, E3, E4) and total crown respiration (R_t) estimated by a uniform R_d and CEZs method in *Prunus lannesiana Wils* in autumn on crown scales. R_{day} and R_t were shown under sunny (closed bar) and cloudy (open bar) conditions, CEZ(D) and CEZ(E) were R_t estimated by using CEZs method respectively under sunny and cloudy conditions. Identical letters indicate homogeneous groups with statistically insignificant differences (p > 0.05). *n*=6.

Fig. S13 The independent (closed bar) and joint (open bar) explanatory variance of ecological factors for hourly R_d in autumn in each CEZ during sunny (a,c,e,g) and cloudy (b,d,f,h) conditions as estimated by hierarchical partitioning, in which '*' denotes that the independent effect due to this variable was significant at p<0.05.

Fig. S14 The independent (closed bar) and joint (open bar) explanatory variance of the factors for spatial R_d in autumn during sunny (a) and cloudy (b) conditions, as estimated by hierarchical partitioning, in which '*' denotes that the independent effect due to this variable was significant at p< 0.05.

Table S1 Morphological characters of the three selected *Prunus lannesianas Wils* with different crown structures in the study

Table S2 Maximal (MAX), average (AVE) and minimal (MIN) values of wind speed, sea level pressure and water vapor pressure of the study site during the two sky conditions.

Table S3 One-way ANOVA in R software (the confidence band with 95%) to

calculate the significant differences of *I* (μmol m^{-2} s^{-1}) during 5:00 to 19:00 among the crown areas of C1-C10.

Table S4 One-way ANOVA in R software (the confidence band with 95%) to calculate the significant differences of *T* (^{o}C) during 8:00 to 16:00 of a day among the crown areas of C1-C10.

Table S5 One-way ANOVA in R software (the confidence band with 95%) to calculate the significant differences of *T* (^{o}C) during 17:00 to 7:00 of a day among the crown areas of C1-C10.

Table S6 One-way ANOVA in R software (the confidence band with 95%) to calculate the significant differences of *T* (^{o}C) during 8:00 to 16:00 of a day among the sub-areas of s1, s2, s3, and s4.

Table S7 One-way ANOVA in R software (the confidence band with 95%) to calculate the significant differences of *RH* (%) among the crown areas of C1-C10.

Table S8 One-way ANOVA in R software (the confidence band with 95%) to calculate the significant differences of C_{air} (mmol mol^{-1}) among the crown areas of C1-C10.

Fig.S1 to Fig. S14:

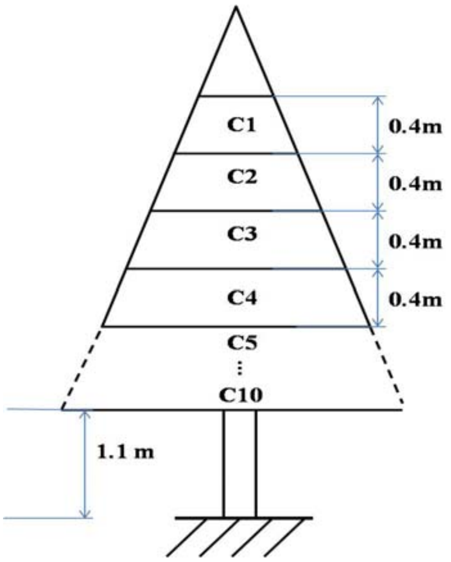

Fig. S1 One of the tree profiles for vertical observation of ecological factors and leaf structural traits on a crown scale.

1) Divisions of crown areas of the studied trees: The crown of approximately 4 m in height was taken as the survey area based on the leaf structural parameters and vertical variations of ecological factors, taking into account the precision and resolution of the detectors. Few newly grown branches and immature leaves at the top of the crown were neglected for survey because of their minimal function for the whole crown, although the entire area was 0.4-0.6 m tall.

2) The criterion of least possible separation distance: The sensitivity of the optic spectrometer (AvaSpec-ULS2048XL, Avantes, Netherlands) was 0.01 $W \cdot m^{-2}$ in a measurement mode of absolute irradiance. The quantitative range for any measurement with a value of less than 0.01 $W \cdot m^{-2}$ was not considered to be an effective result when the vertical division of a crown was less than 0.4 m tall. Therefore, we reasonably divided the spatial areas of a crown by a minimum value of 0.4 m. In this way, each area we divided contained sufficient leaves for measurement and met the observable requirement as the criterion of the least possible separation distance.

3) Representativeness of tree profiles for a 3-dimensional crown: The profile of a crown was divided into ten areas equally in a vertical direction; each area (labelled as C1, C2, C3, C4, C5, C6, C7, C8, C9, C10) was 0.4 m tall, which was three or four times the length of a piece of leaf. Then, six random profiles of a tree crown were sampled and averaged to represent a 3-dimensional tree crown in our study.

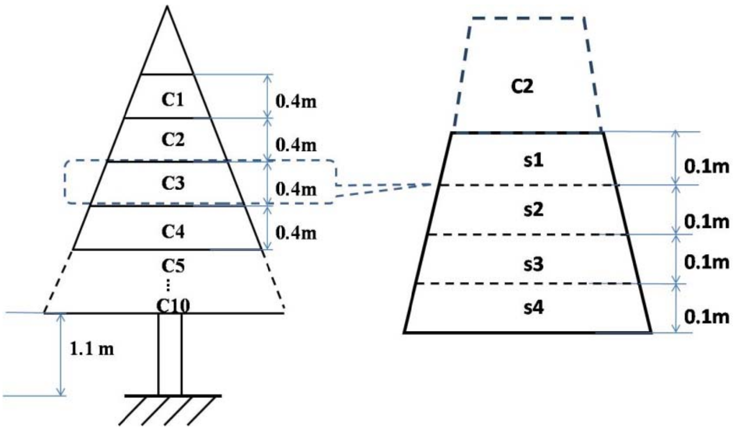

Fig. S2 One of the tree profiles for vertical observation of temperature (*T*) in the area of C3 on a crown scale.

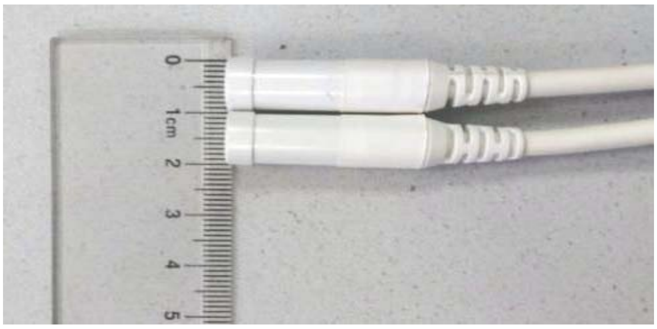

Figure S3 Diameter of temperature sensors

1) **Reasons for dividing C3 area into sub-areas** There were significant differences between each pair of C1, C2, and C3, and there was no significant difference between each pair of C3 to C10. Because the sensitivity of the data loggers (Onset HOBO Data Loggers, Pocasset, Massachusetts, USA) was 0.21 ^{o}C, observational accuracy could be higher in air temperature investigation. Thus, we divided C1, C2, C3 into sub-areas. There was no significant difference between sub-areas in C1 and C2.

2) **The criterion of least possible separation distance in the area of C3** The sensitivity of the data loggers (Onset HOBO Data Loggers, Pocasset, Massachusetts, USA) was 0.21 ^{o}C in a measurement mode of temperature; thus, the quantitative range for any measurement of temperature with a value of less than 0.21 ^{o}C was not

considered to be a significant figure. According to our preliminary experiments with the least measurement distance, when the vertical division of a crown was less than 0.1 m tall, the change of air temperature was less than 0.21 ^{o}C. Therefore, the area of C3 was divided into four sub-areas (labelled as s1, s2, s3, s4) equally, and each sub-area was 0.1 m tall.

3) **Least measurement distance** The diameter of a sensor (Onset HOBO Data Loggers) is 0.01m, locating two sensors side-by-side was considered to be responsive in 0.02 m distance (Figure S3). Thus, the least measurement distance was 0.02 m in the experiment.

4) **Describing variations in sub-areas** Based on our repeated observations in the sub-areas, the significant differences of temperature between each pair of sub-areas steadily appeared in specific period. Although the sub-dividing areas by 0.1m was beyond the level that most crown models would require, and undetectable in field data collections, there were obvious spatial variations of some ecological parameters, it was worth to investigate further. Thus, we explained the sub-area carefully in the text.

5) Because of limited precision and resolution of the fiber optic spectrometer (AvaSpec-ULS2048XL, Avantes, Netherlands), the diurnal dynamics of light intensity on the scale of sub-areas could not be measured accurately. Additionally, the same technical limitations of Onset HOBO Data Loggers led to inaccurate values of observation within a vertical distance of 0.1 m. There was no spatial heterogeneity in a vertical direction for variables of air relative humidity (*RH*) and CO_2 concentration (C_{air}) on the crown scale, so they were not measured on the scale of sub-areas.

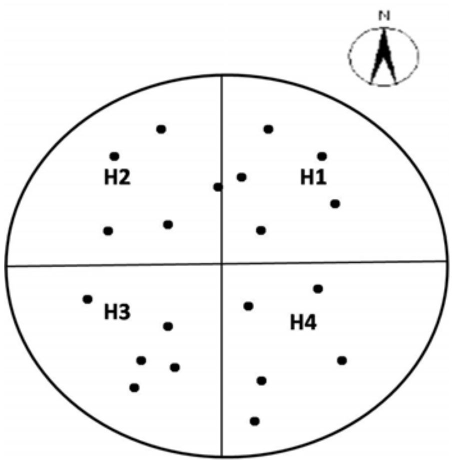

Fig. S4 One of the top views of horizontal observation of ecological factors in each vertical area on a crown scale.

The data from twenty random samples were collected and uniformly distributed across four quadrants (H1, H2, H3, H4) in each vertical area, which were divided by the east-west axis and the north-south axis. The same methods of data collection were used in Fig.S2 and S1.

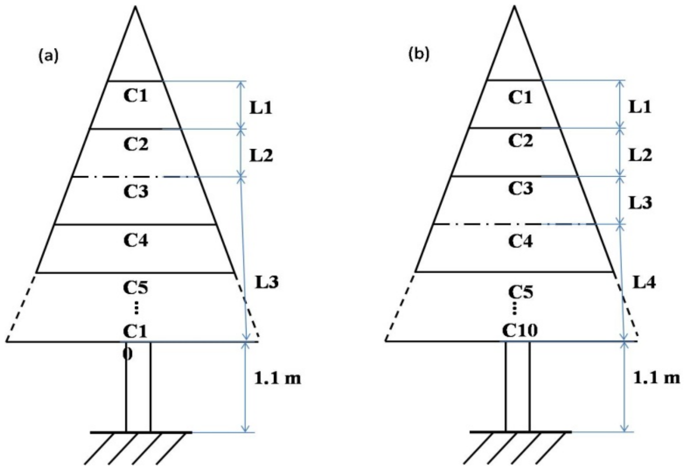

Fig. S5 An ecological zoning for a spatial gradient pattern in a vertical direction for the photosynthetic photon flux density (*I*) on a crown scale under sunny (a) and cloudy (b) conditions.

The statistical significances of *I* appeared between each pair of the areas of C1, C2 and C3 from7:00 to 18:00 during sunny conditions and each pair of the areas of C1, C2, C3 and C4 during cloudy conditions ($p<0.05$) (Table S3). When there were no

significant differences of *I* between two adjacent areas, we considered the two areas as the same CEZ. Thus, the whole crown was divided into three CEZs (C1, C2 and C3-C10) during sunny conditions and four CEZs (C1, C2, C3 and C4-C10) during cloudy conditions. Hence, the spatial heterogeneity of vertical variations in *I* appeared a 3-CEZ (L1, L2, L3) gradient pattern during sunny conditions (a) and a 4-CEZ (L1, L2, L3, L4) gradient pattern (b) during cloudy conditions.

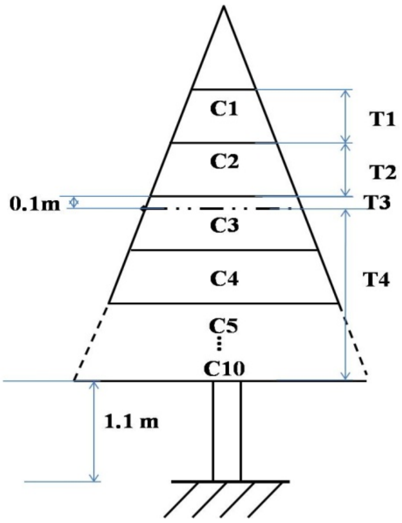

Fig. S6 An ecological zoning for a spatial gradient pattern in a vertical direction for variations of temperature (*T*) on a crown scale.

1) From 8:00 to 15:00 during sunny conditions, there were statistically significant differences in temperature (*T*) between each pair of the areas of C1, C2, and C3, but no significant differences among the areas of C3 to C10 (Table S4).There was no significant difference in *T* during the rest of the day during sunny conditions or the whole day during cloudy conditions (Table S4 and Table S5).

2) From 8:00 to 15:00 during sunny conditions, there were statistically significant differences in *T* between s1 and the other sub-areas but no significant differences between each pair of s2, s3, and s4 (Table S6). If there were no significant differences in *T* between two adjacent areas (sub-areas), we considered the two areas (sub-areas) to be classified as a crown ecological zone (CEZ). Thus, the whole area of

a crown was divided into three CEZs as C1, C2 and C3-C10 by their statistical significance of differences, and the area of C3 was divided into two CEZs (s1 and s2-s4) by synthesizing the analysis results of the statistical significance of differences among areas and sub-areas. The spatial variation of *T* on a crown scale assumed a 4-CEZ gradient pattern as T1, T2, T3 and T4.

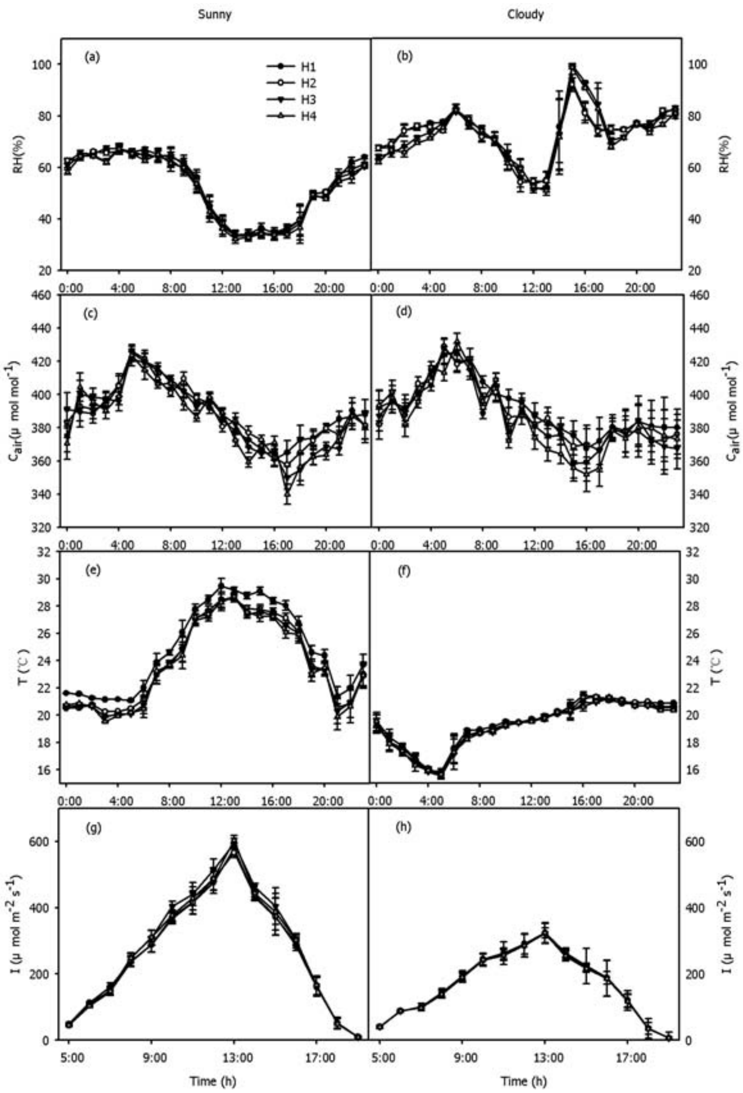

Fig. S7 Hourly average values of *RH* (a,b), C_{air} (c,d), *T* (e,f) and *I* (g,h) for the four crown horizontal quadrants(H1, H2, H3, H4) in one of vertical areas during sunny (a,c,e,g) and cloudy (b,d,f,h) conditions in summer. Each value was the average of five crown samplings from the same quadrant. All values were means ± SE. *n*=5.

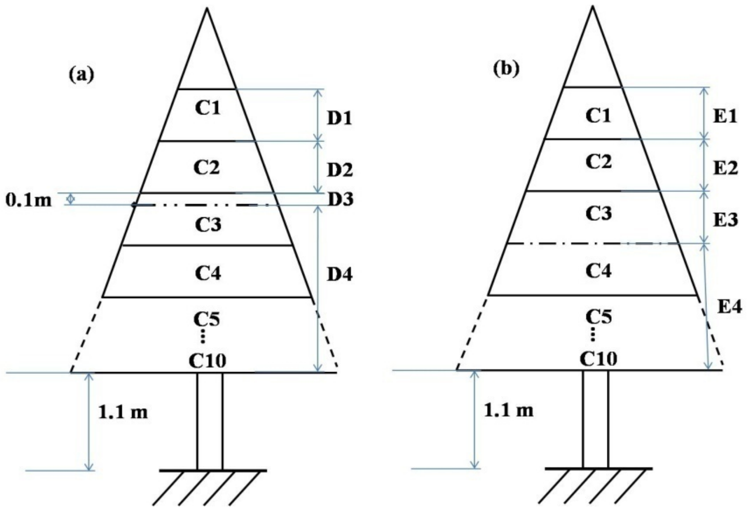

Fig. S8 Ecological zoning integration of ecological factors for spatial gradient patterns on a crown scale under sunny (a) and cloudy (b) conditions.

1) During sunny conditions, the spatial heterogeneity of *T* was integrated as a 4-CEZ gradient pattern (T1, T2, T3, T4) and that of *I* was integrated as a 3-CEZ gradient pattern (L1, L2, L3). The areas of T1 and T2 were consistent with those of L1 and L2, respectively, and the area of L3 was divided into the areas of T3 and T4 by the variable temperature. Thus, the spatial patterns of crown ecological factors were consistent with the gradient pattern of *T*, which was a 4-CEZ gradient pattern (D1, D2, D3, D4) during sunny conditions.

2) During cloudy conditions, the spatial heterogeneity of *I* was a 4-CEZ gradient pattern (L1, L2, L3, L4), but no spatial heterogeneity of *T* existed. Thus, the spatial heterogeneity of the crown ecological factors was consistent with the gradient pattern of *I* during cloudy conditions and was also a 4-CEZ gradient pattern (E1, E2, E3, E4).

3) Consequently, there were significant differences of *T* and *I* between each pair of CEZs during sunny and cloudy conditions, but there was no significant difference in any spatial positions within each CEZ.

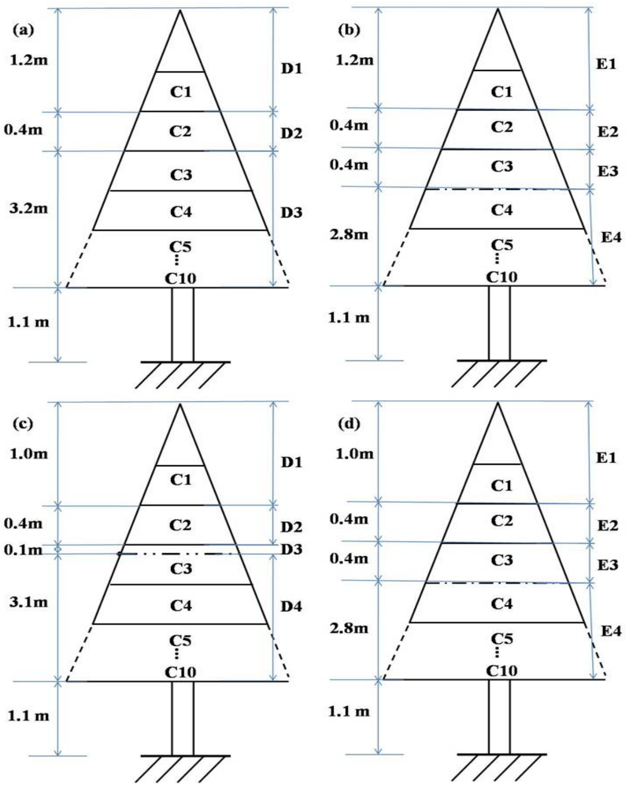

Fig. S9 The CEZs gradient patterns of *Prunus lannesiana Wils* in spring (a,b) and autumn (c,d). The CEZs of the tree were shown under sunny (a,c) and cloudy (b,d) conditions separately.

1) The CEZs gradient pattern in autumn was studied by the above mean during October to November in the same year of summer investigation, while the CEZs gradient pattern in spring was studied by the same mean during May to June in the next year. The CEZs gradient patterns of the rest time of a year were not investigated, because there were few developed leaves on the trees.

2) In spring, it appeared a 3-CEZs pattern (D1, D2, D3) during sunny conditions (a), and a 4-CEZs pattern (E1, E2, E3, E4) during cloudy conditions (b). In autumn, it appeared the same gradient patterns as that in summer, respectively during sunny and cloudy conditions (c,d).

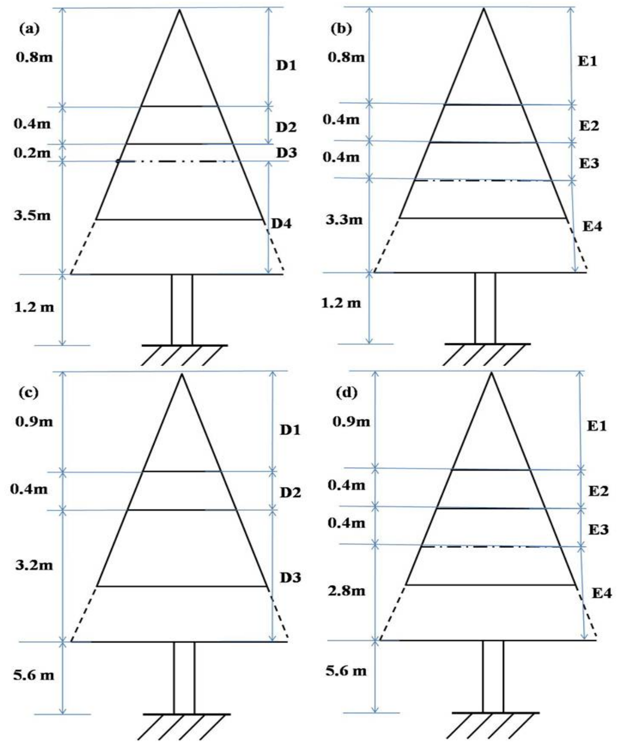

Fig. S10 The CEZs gradient patterns of *Prunus cerasifera* (a,b) and *Koelreuteria paniculata* (c,d) in summer. The CEZs of the tree were shown under sunny (a,c) and cloudy (b,d) conditions separately.

1) The two species were selected in the same location with the *Prunus lannesiana Wils* (Tree-1), and the CEZs gradient patterns were studied by the same mean of Tree-1.

2) The CEZs gradient pattern of *Prunus cerasifera* was similar with *Prunus lannesiana Wils*: a 4-CEZs pattern (D1, D2, D3, D4) during sunny conditions (a) and another 4-CEZs pattern (E1, E2, E3, E4) during cloudy conditions (b). The CEZs gradient pattern of *Koelreuteria paniculata* was a 3-CEZs pattern (D1, D2, D3) during sunny conditions (c) and a 4-CEZs pattern (E1, E2, E3, E4) during cloudy conditions (d).

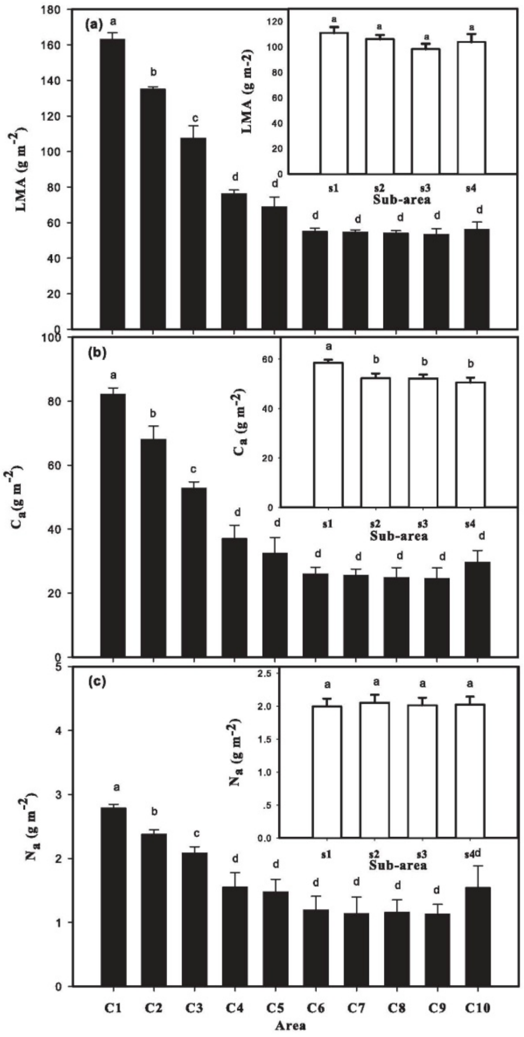

Fig. S11 Vertical variations among different crown areas (closed bar) and sub-areas (open bar). Identical letters indicate homogeneous groups with statistically insignificant differences (p>0.05). All values are means ± SE; *n*=30.

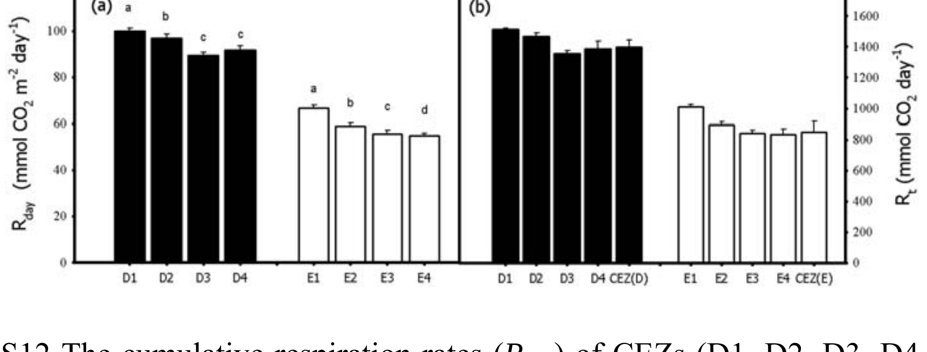

Fig. S12 The cumulative respiration rates (R_{day}) of CEZs (D1, D2, D3, D4, and E1, E2, E3, E4) and total crown respiration (R_t) estimated by a uniform R_d and CEZs

method in *Prunus lannesiana Wils* in autumn on crown scales. R_{day} and R_t were shown under sunny (closed bar) and cloudy (open bar) conditions, CEZ(D) and CEZ(E) were R_t estimated by using CEZs method respectively under sunny and cloudy conditions. Identical letters indicate homogeneous groups with statistically insignificant differences (p > 0.05). *n*=6.

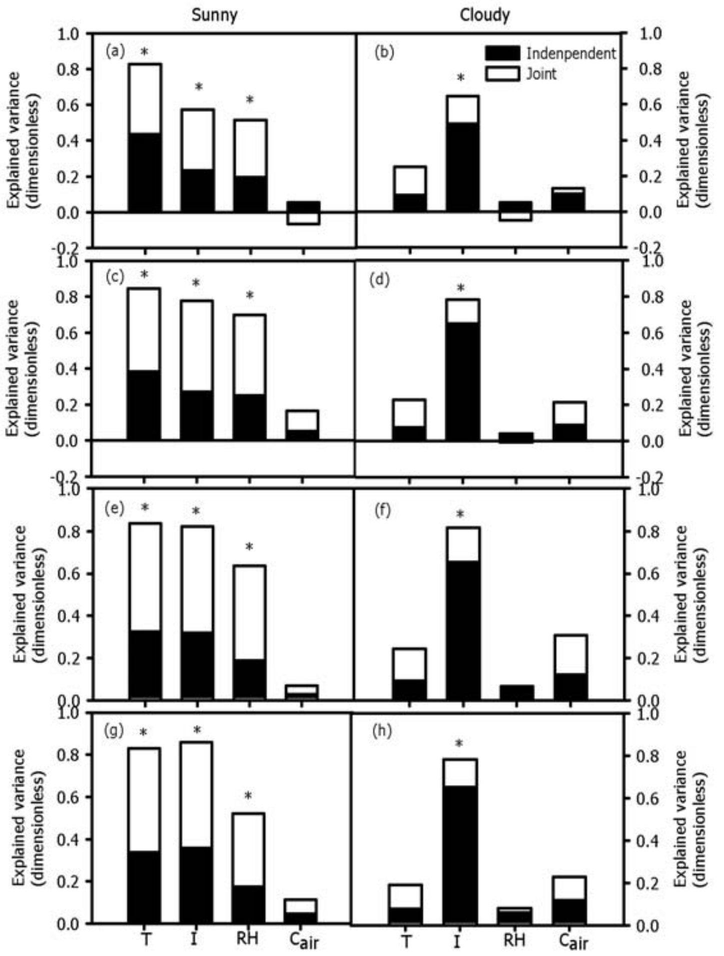

Fig. S13 The independent (closed bar) and joint (open bar) explanatory variances of ecological factors for hourly R_d in autumn in each CEZ during sunny (a,c,e,g) and cloudy (b,d,f,h) conditions as estimated by hierarchical partitioning, in which '*' denotes that the independent effect due to this variable was significant at p<0.05.

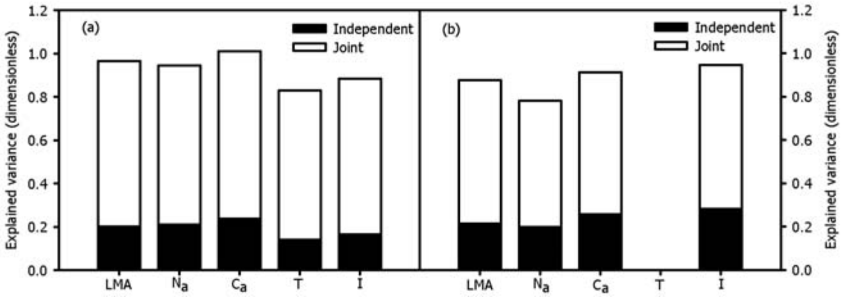

Fig. S14 The independent (closed bar) and joint (open bar) explanatory variances of the factors for spatial R_d in autumn during sunny (a) and cloudy (b) conditions, as estimated by hierarchical partitioning, in which '*' denotes that the independent effect due to this variable was significant at $p< 0.05$.

Table S1 to Table S2

Table S1 Morphological characters of the three selected *Prunus lannesianas Wils.* with different crown structures in the study

	Tree height (m)	Canopy height (m)	Leaf area index ($m^2 \cdot m^{-2}$)	Diameter at breast height (m)	The maximum horizontal diameter (m)
Tree-1	5.7	4.6	4.5	1.213	3.58
Tree-2	5.4	4.5	5.8	0.988	3.60
Tree-3	6.1	4.4	7.1	1.445	3.89

Table S2 Maximal (*MAX*), average (*AVE*) and minimal (*MIN*) values of wind speed, sea level pressure and water vapour pressure of the study site during the two sky conditions.

	Wind speed (m/s)			Sea level pressure (kpa)			Water vapor pressure (kpa)		
	MAX	*AVE*	*MIN*	*MAX*	*AVE*	*MIN*	*MAX*	*AVE*	*MIN*
Sunny	3.45±0.84	1.98±0.42	0	100.54±0.66	100.34±0.66	100.10±0.66	2.56±0.73	1.51±0.64	0.73±0.12
Cloudy	3.99±0.94	1.9±0.41	0	100.32±0.32	100.15±0.33	99.96±0.35	3.28±0.87	2.59±0.36	1.69±0.45

All values are means± SE; *n*=60
